# Meta-analysis of associated factors of sleep disorders in elderly patients with coronary heart disease

**DOI:** 10.3389/fpsyt.2026.1834527

**Published:** 2026-08-10

**Authors:** Siling Tan, Simin Tan, Gufen Jiang, Hua He

**Affiliations:** 1 Department of Nursing, The Second Affiliated Hospital of Hunan University of Chinese Medicine, Changsha, Hunan, China; 2 Department of General Basic Courses, Hunan Defense Industry Polytechnic, Xiangtan, Hunan, China

**Keywords:** associated factors, coronary heart disease, meta-analysis, sleep disorders, systematic review

## Abstract

**Background:**

This meta-analysis aimed to identify the factors associated with sleep disorders in elderly patients with coronary heart disease.

**Methods:**

All eligible published studies were retrieved via comprehensive manual and electronic searches across eight mainstream Chinese and English databases, including PubMed, Embase, Web of Science, the Cochrane Library, CBM, CNKI, Wanfang Database, and VIP. The search period covered all records from the establishment of each database up to January 1, 2026. Two independent researchers separately performed literature screening, methodological quality evaluation, and data extraction to avoid mutual interference. All quantitative pooled analyses were conducted using Stata 14.0 statistical software.

**Results:**

This study ultimately incorporated 22 eligible studies involving 19, 454 elderly patients with coronary heart disease (CHD). Among these enrolled studies, 9 were included for quantitative pooled analysis. Pooled estimates demonstrated significant associations between multiple clinical and demographic factors and sleep disorders in this elderly CHD patient population. Specifically, meta-analysis results showed that diabetes (OR = 1.24, 95% CI: 1.08–1.43), female sex (OR = 2.32, 95% CI: 1.71–3.13), low monthly household income (OR = 3.21, 95% CI: 2.12–4.84), and chronic gastritis (OR = 2.17, 95% CI: 1.54–3.05) were significant risk factors for sleep disorders (all p < 0.05).

**Conclusion:**

Sleep disorders are a critical comorbidity in elderly patients with coronary heart disease, underscoring the urgent need to explore the underlying factors linked to such sleep disturbances. Healthcare administrators can leverage these findings to optimize relevant prevention and management strategies. Identification of high-risk individuals and implementation of targeted interventions enables clinicians to effectively reduce the incidence and severity of sleep disturbances in this CHD patient population.

**Systematic review registration**: https://www.crd.york.ac.uk/PROSPERO/, identifier CRD420251117697.

## 1 Introduction

Coronary heart disease (CHD) is caused by coronary atherosclerotic lesions that result in vascular luminal narrowing, occlusion, or vasospasm. These pathological alterations induce myocardial ischemia, hypoxia, and even myocardial necrosis. The clinical subtypes of CHD mainly consist of myocardial infarction, angina pectoris, and silent myocardial ischemia (1). Patients with CHD frequently experience fatigue, decreased exercise tolerance, blood pressure fluctuations, and increased susceptibility to anxiety. Sleep disorders also substantially promote the onset and progression of CHD, with primary manifestations including insufficient sleep duration, impaired sleep quality, and circadian rhythm disturbances (2). The International Classification of Sleep Disorders, 3rd Edition (ICSD-3), establishes standardized classification and diagnostic criteria for multiple sleep disorder subtypes, including insomnia (3).

Current evidence shows that the prevalence of insomnia among older adults (aged ≥ 60 years) with CHD ranges from 20% to 60%. Nevertheless, comprehensive meta-analyses focusing on the factors associated with sleep disorders in this specific population remain scarce. Accordingly, this meta-analysis was performed to update the evidence regarding factors linked to sleep disorders in elderly CHD patients and provide evidence-based references for clinicians to formulate targeted prevention and management strategies.

## 2 Methods

This systematic review and meta-analysis was performed in strict accordance with the Preferred Reporting Items for Systematic Reviews and Meta-Analyses (PRISMA) guidelines. The study protocol was prospectively registered with the PROSPERO database (Registration No. CRD420251117697).

### 2.1 Search strategy

A systematic literature search was performed to retrieve eligible studies exploring factors associated with sleep disorders in elderly patients with coronary heart disease (CHD). Eight electronic databases, including PubMed, Web of Science, Embase, the Cochrane Library, VIP, China National Knowledge Infrastructure (CNKI), Chinese Biomedical Literature Database (CBM), and Wanfang Data, were searched from database inception to January 1, 2026. Furthermore, manual screening of the reference lists of all enrolled articles was performed to retrieve additional potentially relevant studies. The search strategy employed both MeSH terms and free-text keywords related to coronary heart disease and sleep disorders. Key search terms included “Coronary Disease,“ “Coronary Heart Disease,“ “Myocardial Ischemia,“ “Insomnia,“ “Sleep Disorders, “ “Risk Factors, “ and “associated factors.” The full search strategy used for PubMed is provided in **Table 1**.

**Table 1:** PubMed search retrieval strategy.

Number	Search terms
#1	“Coronary Disease”[MeSH Terms]
#2	((((((((Disease, Coronary[Title/Abstract]) OR (Coronary Heart Disease[Title/Abstract])) OR(Disease, Coronary Heart[Title/Abstract])) OR (Heart Disease, Coronary[Title/Abstract]))OR(Myocardial Ischemia[Title/Abstract])) OR (Acute Myocardial Infarctio[Title/Abstract])) OR (Coronary Artery Disease[Title/Abstract])) OR (Cardiovascular Disease[Title/Abstract])) OR (Angina Pectoris[Title/Abstract])
#3	#1 OR #2
#4	“Sleep Wake Disorders”[Mesh]
#5	((((((Disorder, Sleep Wake[Title/Abstract]) OR (Wake Disorder, Sleep[Title/Abstract])) OR (Disorder, Sleep[Title/Abstract])) OR (Sleep Disorde[Title/Abstract])) OR (Dyssomnias[Title/Abstract])) OR (Insomnia[Title/Abstract])) OR (Sleep Disturbance[Title/Abstract])
#6	#4 OR #5
#7	“Risk Factors”[MeSH Terms]
#8	((effect factor[Title/Abstract]) OR (associated factor[Title/Abstract])) OR (contributing factor[Title/Abstract])
#9	#7 OR #8
#10	#3 AND #6 AND#9

### 2.2 Inclusion and exclusion criteria

#### 2.2.1 Inclusion criteria

- Study design: Cohort, case-control, or cross-sectional studies;
- Participants: Elderly patients (operational definition: mean age ≥ 60 years or a study population predominantly aged ≥ 60 years) diagnosed with CHD and comorbid sleep disorders. Patients with primary sleep disorders were excluded. CHD diagnosis was based on the 2019 European Society of Cardiology guidelines, and sleep disorders were defined according to the American Academy of Sleep Medicine^’^s International Classification of Sleep Disorders (ICSD) criteria;
- Outcome measures: Associated factors of sleep disorders among CHD patients, requiring clearly defined assessment approaches. Inclusion required the availability of odds ratios with their corresponding 95% confidence intervals (CIs), or sufficient raw data for their calculation.

#### 2.2.2 Exclusion criteria

- Studies with unobtainable full texts; reviews, conference abstracts, letters, comments, or study protocols;
- Duplicate publications;
- Articles published in languages other than Chinese or English;
- Studies with low methodological quality.

### 2.3 Data extraction

All retrieved literature records were imported into EndNote 21 to remove duplicate entries. Thereafter, two researchers independently screened the titles and abstracts of the remaining records to select studies that satisfied the predefined inclusion criteria. Full texts of potentially eligible studies were retrieved and evaluated according to pre-established standards for final inclusion. Data extraction was independently conducted by two researchers, followed by mutual cross-checking. The extracted information from each enrolled study included the first author, publication year, study country, sample size, study design, characteristics of the CHD population, sleep assessment tools, mean age or the proportion of participants aged ≥ 60 years, and factors associated with sleep disorders in elderly patients with CHD. Any discrepancies arising during literature screening and data extraction were resolved via mutual discussion. If consensus could not be achieved, a third senior researcher was consulted to reach a final adjudication.

### 2.4 Quality appraisal

Two researchers independently evaluated the methodological quality of all enrolled studies. The 11-item quality assessment tool established by the Agency for Healthcare Research and Quality (AHRQ) was utilized for the quality evaluation of included cross-sectional studies. Each item was assessed as “yes”, “no”, or “unclear”, with total scores ranging from 0 to 11. According to the aggregate score, the included studies were categorized into three quality grades: low quality (0–3 points), moderate quality (4–7 points), and high quality (8–11 points).

The methodological quality of cohort and case-control studies was evaluated using the Newcastle-Ottawa Scale (NOS). The NOS comprises eight items across three core domains: study group selection, inter-group comparability, and outcome (or exposure) ascertainment, with a maximum total score of 9. Specifically, studies with a score of ≥7 were defined as high quality, whereas those with lower scores were categorized as low quality. Any discrepancies between the two reviewers were resolved via mutual discussion. If no consensus was achieved, a third senior reviewer adjudicated the final outcome.

The certainty of evidence for primary outcomes was evaluated using the Grading of Recommendations Assessment, Development and Evaluation (GRADE) framework. Evidence certainty was classified into four grades: high, moderate, low, and very low, according to five key domains, including risk of bias, inconsistency, indirectness, imprecision, and publication bias.

### 2.5 Statistical analysis

All extracted data and methodological quality assessment results were imported into Microsoft Excel 2021 for standardized data management. All statistical analyses were conducted using Stata 14.0 software. Quantitative meta-analysis was limited to sleep disorder-related factors measured via the Pittsburgh Sleep Quality Index (PSQI), with a minimum of three eligible studies required for pooling. Variables with fewer than three studies, outcomes assessed using non-PSQI tools, obstructive sleep apnea (OSA)-related indicators, and factors with substantial inter-study heterogeneity were analyzed via descriptive summary instead of quantitative pooling. Effect sizes were calculated as odds ratios (ORs) with corresponding 95% confidence intervals (CIs). Between-study heterogeneity was evaluated using the chi-square (Q) test and I² statistic. Given that only three to four studies were included for each pooled factor, leave-one-out analysis was not performed; instead, sensitivity assessment was completed via alternating fixed-effect and random-effects models to judge the stability of pooled results. For outcomes with unstable pooled estimates or persistent unexplained heterogeneity even after sensitivity evaluation, we adopted qualitative narrative summary rather than quantitative meta-analytic pooling.

Publication bias was assessed using Egger’s test for factors with at least ten included studies. Factors with fewer than ten studies were not assessed for publication bias. Statistical significance was defined as a two-sided P < 0.05.

## 3 Results

### 3.1 Study process

**Figure 1:**
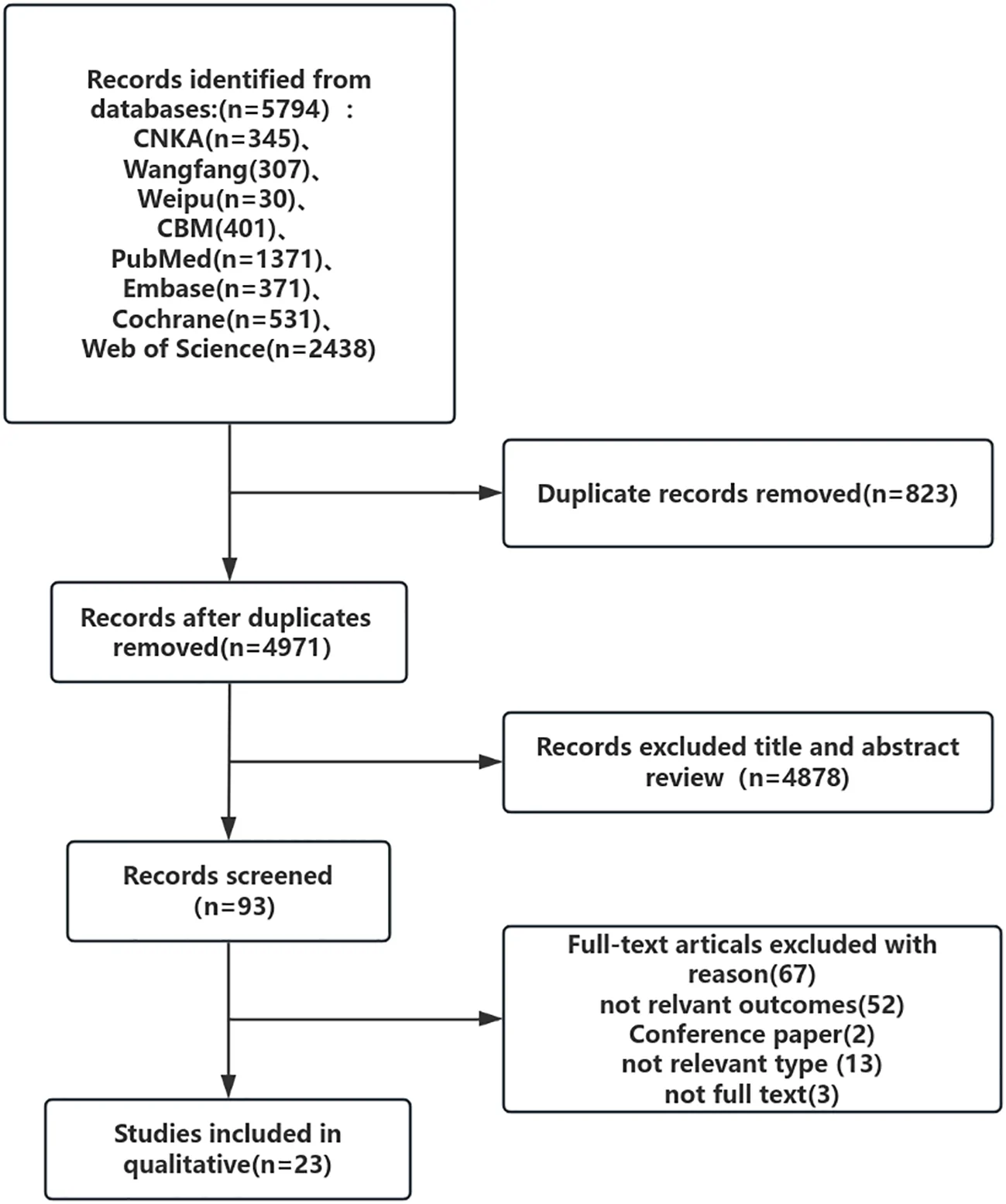
PRISMA flow chart: illustration of the study inclusion and exclusion process. n denotes the count.

A preliminary search identified 5, 794 records. After importing the results into EndNote 21 and removing duplicates, 4, 971 records remained. Following title and abstract screening, 4, 878 records were excluded. The full texts of the remaining records were retrieved and assessed for eligibility, and 71 studies that did not meet the inclusion criteria were excluded. Ultimately, 22 studies were included in the meta-analysis, comprising 20 cross-sectional studies, 1 cohort study, and 1 case-control study. The study selection process is detailed in **Figure 1**.

### 3.2 Characteristics and quality of included studies

Twenty-two studies (4–25) published between 2016 and 2025 were included, involving a total of 19, 454 participants from six countries: Australia, Lithuania, Sweden, Norway, India, and China. Quality assessment using the AHRQ scale revealed that among the included cross-sectional studies, 16 (4, 5, 7, 9–12, 14, 15, 17–21, 23, 24) scored 5–7 points and were classified as moderate quality, while 4 (8, 13, 16, 22) scored 8–11 points and were classified as high quality. The remaining cohort and case-control studies (6, 25) were evaluated using the Newcastle-Ottawa Scale (NOS), both demonstrating moderate quality (scores 5–6). **Table 2** presents the baseline characteristics and methodological quality of the included studies. We further evaluated the reliability of the pooled associations using formal evidence grading. The GRADE approach was employed to assess the certainty of evidence for the associated factors included in the analysis. We graded the certainty of evidence for the four risk factors using the GRADE approach. For female sex, lower monthly household income and chronic gastritis, evidence was rated as very low certainty. This downgrade occurred mainly because the included studies carried substantial methodological bias risks. For diabetes, we found no critical flaws across risk of bias, inconsistency, indirectness and imprecision domains, so its overall evidence certainty remained low. We could not test for publication bias for any of the four associations, given that each analysis pooled fewer than 10 studies. Full GRADE assessment results are available in **Supplementary File 1**.

**Table 2:** General profiles of eligible literature.

Study	Nation	Year	Sample size	study design	CHD population	Mean age/Proportion ≥60 years	Sleep assessment tool (PSQI cut-off)	Risk factors	Quality score(point)	
									NOS	AHRQ
Cao (4)	China	2025	164	A	CAD	-/70%	PSQI>7	1.2.3.4.5.6		5
Chen (5)	China	2025	168	A	AMI	-/73%	PSQI≥7	7.8.9.10		5
Dong (6)	China	2016	262	B	CAD	64.97	PSQI>7	2.3.4.11.12.13.14	5	
He (7)	China	2024	515	A	CAD	60 ± 12.7	PSQI>7	1.3.15		7
Hu (8)	China	2025	186	A	PCI	-/68%	PSQI>7	3.16.17.18		9
Lei (9)	China	2020	348	A	CAD	62.6 ± 11.2	PSQI>7	4		5
Mo (10)	China	2025	345	A	CAD	-/65%	PSQI>7	3.4.6.11.19		5
Yu (11)	China	2024	216	A	CAD	61.53	PSQI≥8	20.21.22.23.24.		7
Wang (12)	China	2017	130	A	CAD	-/62%	PSQI>7	3.4.11.25		6
Jing (13)	China	2024	635	A	CAD	-/83%	PSQI>7	26.27.28.29		9
Yang (14)	China	2024	244	A	CAD	68.14 ± 11.72	PSQI>5	3.4.17.19		6
Wang (15)	China	2025	62	A	PCI	64.32	PSQI>7	3.4.30.31		6
Wang (16)	China	2019	102	A	AMI	65.3 ± 7.9	PSQI>7	1.2.5.32.33		9
Wang (17)	China	2024	128	A	PCI	69.95	PSQI>7	1.4.34.35.36.37		5
Wu (18)	China	2021	501	A	CAD	-/62.87%	PSQI>5	11.19.21.46		5
Huang (19)	China	2021	1243	A	CAD	62.17	OSA diagnosis	38		5
Cheng (20)	China	2022	348	A	CAD	62.6 ± 11.2	PSQI>7	3.4		5
Ooi (21)	Australia	2022	9885	A	CAD	64	OSA diagnosis	3.5.13.17.20.43.44		6
Alonderis (22)	Lithuania	2017	772	A	CAD	60 ± 9	Epworth	45		9
Kjellsdotter (23)	Sweden	2020	1718	A	CAD	63.1 ± 9.9	PSQI	39		5
Frøjd (24)	Norway	2021	1082	A	CAD	60.2 ± 9.9	Bergen Insomnia Scale	4.11.17.39.40		6
Muthukrishnan (25)	India	2020	400	C	CABG	-/67%	PSQI>5	4.17.41.42	6	

A, Cross-Sectional Study; B, Case–Control Study; C, Cohort Study; Percutaneous Coronary Intervention (PCI); Acute Myocardial Infarction (AMI); Coronary Artery Bypass Grafting (CABG); CAD, Coronary Artery Disease (stable). Associated factors: 1. advanced age 2. monthly household income 3. depression 4. anxiety 5. heart failure 6. course of coronary heart disease 7. syndrome of TCM 8. NLR 9. FBG 10. triglyceride 11. female sex 12. retirement or resignation 13. hypertension 14. have a late night 15. abstract ability 16. Social support degree 17. diabetes 18. chest pain 19. chronic gastritis 20. male sex 21. health insurance 22. Serum cortisol/testosterone levels 23. gensini’s score 24. angina 25. family dysfunction 26. the environment in the ward is not pleasant 27. severe psychological burden 28. severe illness and discomfort symptoms 29. severe postoperative recovery burden 30. pain at the puncture site 31. take in liquids before going to bed 32. arrhythmia 33. AIMI 34. SOD 35. MDA 36. WHR 37. weekly exercise time 38. MHR 39. type d personality 40. c-reactive protein ≥2 mg/L 41. body mass index >30kg/m ² 42. sedentary lifestyle 43. asthma 44. dyslipidemia 45. left ventricular hypertrophy 46. angina pectoris grade.

For monthly household income, the definition of “low income” was based on the specific cut-off values (<2000 or <3000 RMB) reported in each original study.

### 3.3 Results of meta-analysis

**Figure 2:**
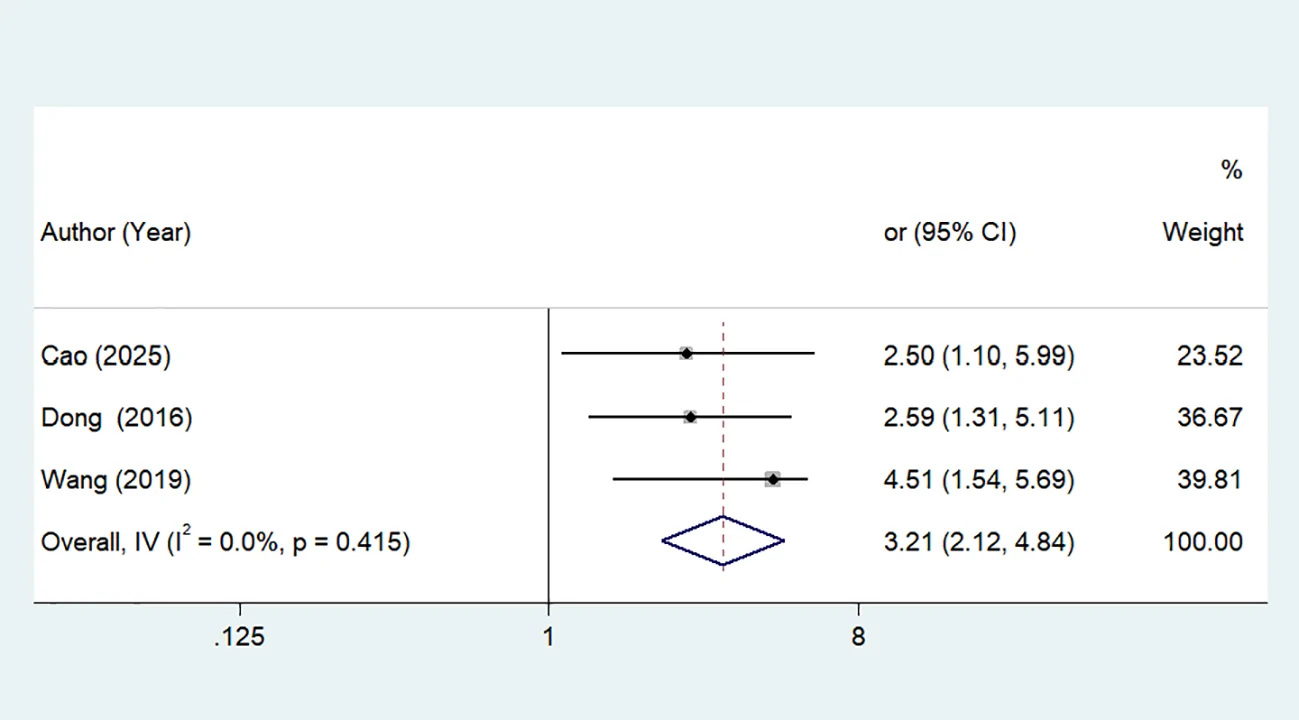
Forest plot illustrating the impact of monthly household income on sleep quality among elderly patients with coronary heart disease.

**Figure 3:**
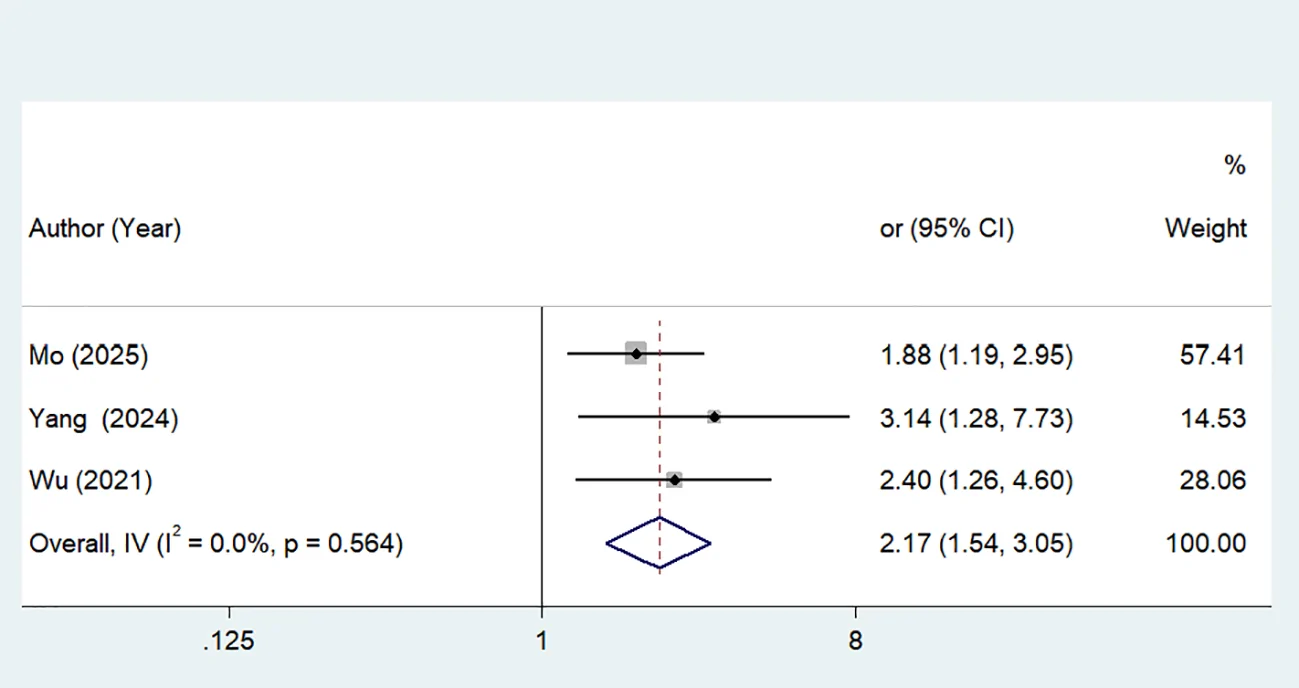
Forest plot illustrating the impact of chronic gastritis on sleep quality in elderly patients with coronary heart disease.

**Figure 4:**
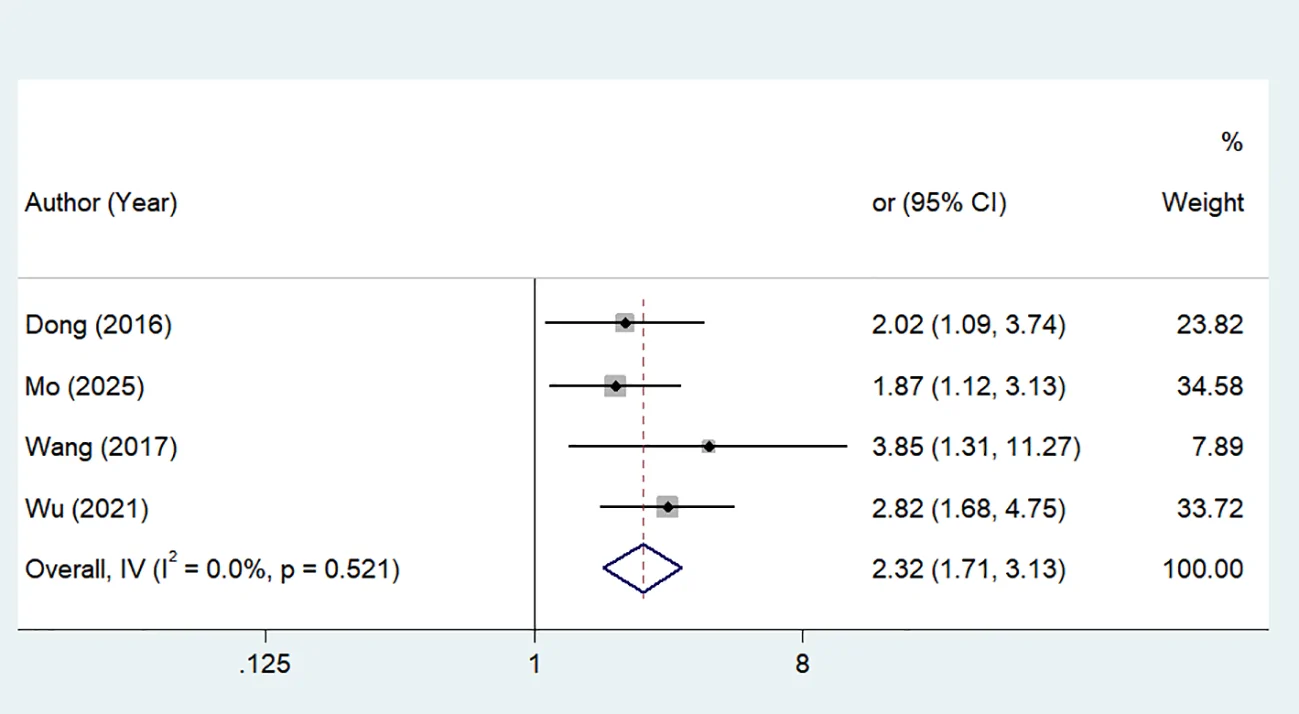
Forest plot illustrating the effect of gender on sleep quality in older patients with coronary heart disease.

**Figure 5:**
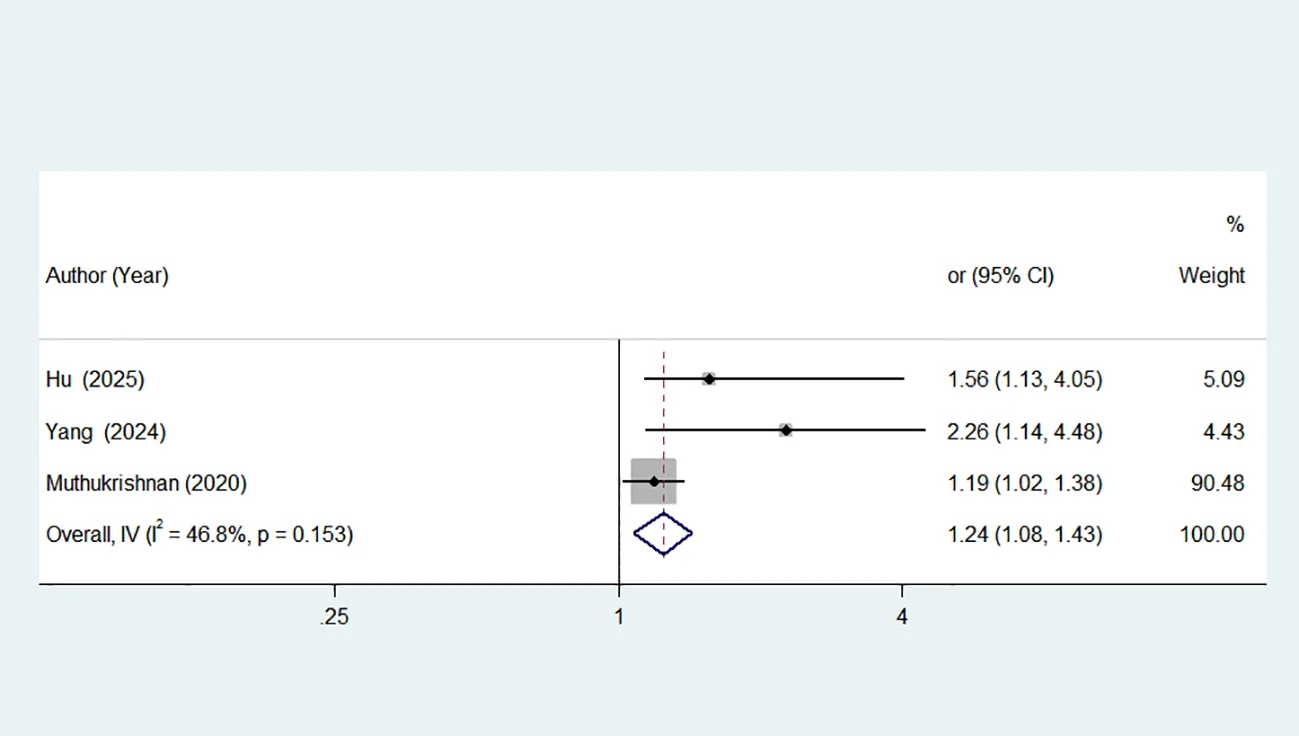
Forest plot illustrating the impact of diabetes on sleep quality in older patients with coronary heart disease.

Of the 22 included studies, 9 (4, 6, 8, 10, 12, 14, 16, 18, 25) were eligible for meta-analysis. Four factors associated with sleep disorders were analyzed: low monthly household income, diabetes, chronic gastritis, and female sex. Pooled odds ratios with 95% confidence intervals were calculated. The results demonstrated that all four factors were significantly associated with sleep disorders (all P < 0.05; **Table 3**): low monthly household income (OR = 3.21, 95% CI: 2.12–4.84; **Figure 2**), chronic gastritis (OR = 2.17, 95% CI: 1.54–3.05; **Figure 3**), female sex (OR = 2.32, 95% CI: 1.71–3.13; **Figure 4**), and diabetes (OR = 1.24, 95% CI: 1.08–1.43; **Figure 5**).

**Table 3:** Pooled meta-analytic estimates for sleep disorder associated factors in elderly patients diagnosed with coronary heart disease.

Risk factors	Heterogeneity test		Test model	Synthesized analysis results				Number of studies
	I^2^ (%)	P		Z	P	OR	95% CI	
diabetes	46.80%	0.153	Fixed	2.947	0.003	1.24	1.08~1.43	3
female gender	0.00%	0.521	Fixed	5.451	<0.001	2.32	1.71~3.13	4
Lower monthly household income	0.00%	0.415	Fixed	5.548	<0.001	3.21	2.12~4.84	3
Chronic Gastritis	0.00%	0.564	Fixed	4.422	<0.001	2.17	1.54~3.05	3

### 3.4 Descriptive analysis

Factors associated with sleep disorders in this study were identified based on the Pittsburgh Sleep Quality Index (PSQI) scale. Obstructive sleep apnea (OSA)—which was not assessed using the PSQI or was subjectively diagnosed—and other associated factors with substantial heterogeneity were excluded from the meta-analysis. For these excluded variables, a qualitative descriptive analysis was employed.

Two studies indicated an association between Type D personality traits and sleep disorders in elderly patients with coronary atherosclerosis. Two other studies identified urban employee medical insurance as a factor associated with sleep disorders. Furthermore, two studies reported a link between the progression of coronary heart disease and sleep disorders. Findings regarding gender were inconsistent: two studies identified an association in men, whereas five studies identified women as a relevant risk factor. Additionally, two studies found a correlation between angina severity and sleep disorders, while two others suggested that heart failure negatively impacts sleep quality. Regarding psychological factors, seven studies reported an association between depression and sleep disorders, although effect sizes varied significantly (OR 1.12–5.61). Nine studies confirmed a link between anxiety and sleep disturbances, but the strength of the association varied across studies, resulting in a wide range of results. Findings regarding the OSA subgroup were also mixed: Ooi’s study indicated that depression is a significant associated factor for OSA-type sleep disorders, while Frøjd suggested that anxiety and female sex are related factors. Both studies consistently confirmed diabetes as an important factor for OSA development. A detailed description of the results for indicators assessed using non-PSQI scales is provided in **Supplementary Material File 2**.

Multiple studies demonstrated that inadequate social support, hypertension, obesity, limited mobility, living environment, arrhythmia, sedentary lifestyle, heart failure, and asthma were associated with sleep disorders. Furthermore, additional research indicated that traditional Chinese medicine (TCM) syndromes, malondialdehyde (MDA), superoxide dismutase (SOD), monocyte/high-density lipoprotein cholesterol ratio (MHR), and triglyceride levels were also linked to sleep disorders.

### 3.5 Sensitivity and publishing bias analysis

Sensitivity analyses were performed by alternately applying fixed-effect and random-effects models to verify the robustness of pooled estimates. Given that only three to four studies were available for each pooled factor, the leave-one-out approach was not implemented, as removing a single study would leave too few remaining studies for reliable re-synthesis. Results were considered robust if effect magnitudes, confidence intervals and statistical significance remained largely consistent between the two analytical models.For female sex, low monthly household income, and chronic gastritis, the pooled ORs, 95% CIs, and P-values remained consistent under both statistical models, demonstrating reliable and robust findings. For diabetes, although minor discrepancies existed in point estimates and statistical significance between the two models, the overall association direction was unchanged; therefore, the corresponding pooled result was still regarded as robust. As fewer than 10 studies were included for each factor, Egger’s test was not performed to assess publication bias. Comprehensive results of the pooled analyses are presented in **Table 4**.

**Table 4:** Sensitivity analysis.

Risk factors	Fixed effect		Random effect		Stability	Egger’s test
	OR (95% CI)	P-value	OR (95% CI)	P-value		
diabetes	1.24(1.08~1.43)	0.003	1.43(1.00~2.07)	0.053	T	—
female gender	2.32(1.71~3.13)	<0.001	2.32(1.71~3.13)	<0.001	T	—
Lower monthly household income	3.21(2.12~4.84)	<0.001	3.21(2.12~4.84)	<0.001	T	—
Chronic Gastritis	2.17(1.54~3.05)	<0.001	2.17(1.54~3.05)	<0.001	T	—

T, Stable.

## 4 Discussion

The population with coronary heart disease (CHD) inherently exhibits clinical heterogeneity, which complicates data analysis. This study included studies involving various CHD subgroups: patients with acute myocardial infarction (AMI), those following percutaneous coronary intervention (PCI), recipients of coronary artery bypass grafting (CABG), and individuals with stable CHD. The etiology of sleep disorders differs fundamentally among these populations; patients in the acute phase or perioperative period often experience severe sleep fragmentation due to physical pain and the intensive care unit environment, whereas those with chronic conditions typically exhibit difficulty maintaining sleep. Furthermore, psychological burden, variations in medication regimens, and varying disease severity significantly impact sleep quality. Consequently, pooling data from patients with such divergent disease trajectories inevitably leads to statistical heterogeneity.

### 4.1 Physiological associated factors

Advanced age is widely recognized as a factor associated with sleep disorders in individuals with CHD. Consequently, the prevalence of sleep disorders is higher among elderly patients with coronary artery disease compared to the general population of patients with this condition (10). Consistent with this, Cao et al. (4) also documented an elevated prevalence of sleep disorders in the elderly CHD population.

Fluctuations in endogenous estrogen levels induce physiological changes that render women more susceptible to sleep disturbances than men (26). Sex is a significant factor influencing sleep disorders in CHD cohorts. The present study demonstrated that female sex was an independent correlate of sleep disorders. However, other studies have reported a higher prevalence of sleep disorders among men compared to women in this population (27). These findings highlight inconsistencies in published literature regarding the effect of sex on sleep disturbances in patients with coronary heart disease.

This study found that elderly patients with coronary heart disease and comorbidities such as diabetes and chronic gastritis had a significantly higher likelihood of sleep disorders compared to those without these conditions. Our pooled analysis results are consistent with findings from previous studies. Furthermore, Svensson et al. (28) identified that diabetes was significantly correlated with sleep disorders. Cantay et al. (29) also identified chronic gastritis as a factor associated with sleep disorders. While Lao et al. (30) reported hypertension as a contributor to poor sleep quality, Parati et al. (31) confirmed that heart failure was also associated with sleep disorders.

### 4.2 Psychological associated factors

Depression and anxiety are significant associated factors for sleep disorders in patients with coronary artery disease, with most studies demonstrating a strong correlation between these psychological issues and poor sleep quality (32, 33). Mechanistically, anxiety increases sympathetic excitability and arousal, hindering sleep maintenance (34), while depression may reduce melatonin secretion, thereby disrupting circadian rhythms and sleep patterns (35). Furthermore, research by Bai et al. (36) indicated that specific sleep quality profiles are associated with an increased risk of developing depressive and anxiety symptoms. Although a clear physiological link exists, a qualitative review of the literature revealed significant heterogeneity in the strength of these associations across the included studies. Given the substantial influence of numerous clinical confounders and differences in study design that were not accounted for, it was considered inappropriate to perform a meta-analysis or subgroup analysis. Consequently, a descriptive analysis was adopted rather than pooling the data to derive a single effect size.

Type D personality is characterized by the co-occurrence of social inhibition and negative affectivity. These traits negatively impact sleep quality through an interplay of worry-related cognitive processes and anxious emotional states (37).

### 4.3 Social associated factors

Post-retirement economic decline among elderly patients with CHD, characterized by reduced personal and household income, is associated with poor sleep quality and a higher likelihood of sleep disorders compared to higher-income groups (11). However, the definition of low income varied slightly across the included studies, with cut-off values of 2, 000 RMB and 3, 000 RMB. Although all included studies focused on Chinese subjects, the study employing the higher threshold (3, 000 RMB) was conducted more recently, likely reflecting rising average income levels over time. Future research should adopt standardized definitions to facilitate cross-regional comparisons. In addition to economic factors, environmental and lifestyle factors also play a significant role. Hospitalization-related environmental disruptions, such as noise and poorly regulated lighting in cardiac care units, are known to adversely affect the sleep patterns of CHD patients (38). Furthermore, sedentary behavior, alcohol consumption, smoking, and excessive tea intake are modifiable lifestyle risk factors for sleep disorders in this population (25, 39–41). Notably, urban medical insurance was found to be protective against sleep disorders; insured patients typically benefit from better financial status and lower out-of-pocket medical expenses, thereby alleviating psychological distress and negative affectivity that may otherwise compromise sleep quality (19).

### 4.4 Other associated factors

Current evidence suggests that medications may adversely affect sleep quality (42). Older patients with coronary heart disease often have multiple comorbidities, necessitating long-term medication use. Research has confirmed an association between certain medications and sleep disorders (43). For instance, diuretics can increase nocturia (42). Antihypertensive agents may trigger insomnia and depressive symptoms (44). Furthermore, antiarrhythmic drugs can induce psychomotor agitation and restlessness (45). The cumulative adverse effects of these medications ultimately compromise sleep quality in this susceptible elderly population.

### 4.5 Strengths and limitations

This study underscores the importance of comprehensive assessments, individualized interventions, and health education for elderly patients with coronary heart disease, while also identifying factors associated with sleep disorders in this population. However, several limitations of this study should be noted: (1) This meta-analysis was restricted to factors reported in the primary studies, which may introduce selection bias into the pooled estimates. (2) The majority of included studies were cross-sectional (n=22), with only one cohort study and one case-control study. Given that 91% of participants were derived from cross-sectional investigations, the observed associations cannot be confirmed as causal and should be regarded merely as correlates. Furthermore, the predominance of this study design, combined with the generally moderate quality of the included studies, limits the ability to infer causality and constrains the overall certainty of the evidence. (3) Subgroup analyses could not be implemented given limited available data in the original studies. We performed sensitivity analyses by alternating fixed-effect and random-effects models to evaluate the stability of pooled estimates rather than identify heterogeneous sources. Any between-study heterogeneity was likely derived from discrepancies in the baseline profiles of CHD participants across trials. (4) Despite comprehensive searches in both Chinese and English databases, the exclusion of unpublished studies or studies in other languages may introduce publication bias, potentially affecting the robustness of our findings. (5) The included studies showed substantial variation in sample sizes, ranging from 62 to 9, 885 participants. This variation may contribute to heterogeneity in the pooled effects and limit the generalizability of the evidence.

## 5 Conclusion

Sleep disorders are a common comorbidity among older adults with coronary heart disease. The present meta-analysis demonstrated that female sex, diabetes, low income, and chronic gastritis were significantly associated with sleep disorders in this population. These findings identify key risk factors and provide a foundation for clinicians to develop targeted interventions aimed at improving sleep quality and overall quality of life. However, these results should be interpreted with caution. We performed GRADE assessment to rate evidence certainty for these associations.Evidence for female sex, low household income and chronic gastritis was downgraded to very low certainty mainly owing to high methodological bias risk within included studies. Evidence for diabetes was graded low certainty with no severe flaws across all evaluation domains. Notably, publication bias could not be evaluated for all pooled indicators owing to the limited number of included studies, which means the real association strength between these risk factors and sleep disorders may be overestimated.

Finally, given that more than 91% of the included studies employed a cross-sectional design, the current findings cannot establish causality between these risk factors and sleep disorders. Therefore, there is an urgent need for rigorous, large-scale, multicenter prospective cohort studies to not only validate these associations and elucidate causal mechanisms but also to enhance the certainty of future evidence assessments.

## 6 Future implication

The findings of this meta-analysis identify several key areas for future research aimed at addressing the limitations of existing studies and further enhancing the reliability of evidence regarding sleep disorders in older adults with CHD.

Primarily, there is an urgent need for well-designed longitudinal studies. In this meta-analysis, 91% of the data were derived from cross-sectional studies; therefore, the factors identified cannot be confirmed as definitive causes of sleep disorders. Future research should prioritize large-scale, prospective cohort investigations to clarify the causal relationships between variables and determine whether these associations are indeed precursors to sleep disorders or merely co-occurring conditions.

Second, a standardized criterion for evaluating socioeconomic status is essential. The income thresholds used to define ‘low income’ in the included studies varied significantly, ranging from 2, 000 to 3, 000 RMB per month. This variation limits the comparability of findings across different regions. It is recommended that future multicenter studies establish a unified income threshold adjusted for inflation. Specifically, criteria could be based on local minimum subsistence standards or purchasing power parity to mitigate confounding effects of long-term inflation and accurately investigate the true relationship between economic hardship and sleep disorders.

## Data Availability

The original contributions presented in the study are included in the article/**Supplementary Material**. Further inquiries can be directed to the corresponding author.
