# Supplementary material for: Meta-analysis of associated factors of sleep disorders in elderly patients with coronary heart disease
Source: Front Psychiatry. 2026 Aug 10;17:1834527. doi: 10.3389/fpsyt.2026.1834527 (PMC13500759; doi:10.3389/fpsyt.2026.1834527)
Supplement: Supplementary file 1 [file Supplementaryfile1.docx]

File 2 Risk factors associated with sleep disorders: studies using assessment tools other than the PSQI

| Study/year | Sleep assessment tool | Risk factors | OR(95%CI) |
| --- | --- | --- | --- |
| Huang^[19]^2021 | OSA diagnosis | severe illness and discomfort symptoms | 1.63(1.06-2.52) |
| Ooi^[21]^2022 | OSA diagnosis | depression | 1.81(1.55-2.12) |
|  |  | heart failure | 1.63(1.22-2.18) |
|  |  | hypertension | 1.61(1.32-1.95) |
|  |  | diabetes | 1.84(1.58-2.14) |
|  |  | male sex | 2.22(1.86-2.65) |
|  |  | asthma | 1.61(1.30-1.96) |
|  |  | dyslipidemia | 1.46(1.22-1.76) |
| Alonderis^[22]^2017 | Epworth | left ventricular hypertrophy | 1.50(1.04-2.20) |
| Frøjd^[24]^2021 | Bergen Insomnia Scale | anxiety | 5.61(3.37-9.35) |
|  |  | female gender | 1.88(1.25-2.83) |
|  |  | diabetes | 1.83(1.17-2.87) |
|  |  | type d personality | 1.69(1.02-2.81) |
|  |  | C-reactive protein ≥2 mg/L | 1.58(1.13-2.22) |
