# Supplementary material for: Meta-analysis of associated factors of sleep disorders in elderly patients with coronary heart disease
Source: Front Psychiatry. 2026 Aug 10;17:1834527. doi: 10.3389/fpsyt.2026.1834527 (PMC13500759; doi:10.3389/fpsyt.2026.1834527)
Supplement: Supplementary file 2 [file Supplementaryfile2.docx]

File 1 GRAED for quality of evidence profile

| outcome | studies | Effect (95% CI) | Risk of bias | Inconsistency | Indirectness | Imprecision | Publication bias | Certainty of evidence |
| --- | --- | --- | --- | --- | --- | --- | --- | --- |
| diabetes | 3 | 1.52(1.11-2.08) | not serious | not serious | not serious | not serious | unable to assess | low |
| female gender | 4 | 2.32(1.71-3.13) | serious | not serious | not serious | not serious | unable to assess | Very low |
| Lower monthly household income | 3 | 3.21(2.12-4.84) | serious | not serious | not serious | not serious | unable to assess | Very low |
| Chronic Gastritis | 3 | 2.17(1.54-3.05) | serious | not serious | not serious | not serious | unable to assess | Very low |
